# Medical mentorship deconstructed: an analysis and structural recommendation for high value mentorship

**DOI:** 10.12688/mep.18944.2

**Published:** 2023-08-30

**Authors:** Duane Kim, Rosa D. Manzo, Michael Montoya, Marissa Nguyen, Kao Houa Vang, Lindsey Weber, Marisela Yepez

**Affiliations:** 1 School of Medicine, University of California Davis Medical Center, Sacramento, CA, USA; 2 Health Sciences Research Institute, University of California, Merced, Merced, CA, USA

**Keywords:** Mentoring, Best evidence medical education, undergraduate, medical education research, qualitative research

## Abstract

*Background: *Mentorship is an important component for young students interested in pursuing a career in medicine. In medically underserved areas, such as rural areas, mentorship can be sparse due to the lack of access to healthcare professionals. The purpose of this project was to gain an understanding of the mentorship received by practicing medical students.

*Methods: *The authors conducted structured, one-on-one interviews with 10 current medical students about their perceptions and experiences with mentorship. Interviews were transcribed, coded, and analyzed for themes and subthemes.

*Results: *Our findings revolve around three time periods of mentorship: 1) Before Obtaining a Mentor; 2) During the Mentorship; and 3) After the Mentorship.  In our findings we describe key characteristics such as professional development, personal qualities of the mentor, and professional and personal guidance as important components in guiding the mentee starting from the undergraduate level and continuing to their current level of education.

*Conclusion: *Interviewees’ experiences with and perspectives on the mentorship they received were generally positive, though it was evident there are some aspects of the mentee-mentor relationship that can be improved. Building on the results obtained, setting expectations, providing mentor training, and pairing mentors/mentees from similar backgrounds are what we propose to create fulfilling and meaningful relationships between a mentee and mentor.

## Introduction

Despite medical school admissions increasing ( Institute of Medicine (US) Division of Health Sciences Policy, 1983), the numbers of prospective doctors are not enough to address the physician shortage, especially in rural areas ( Cooper, 2004; U.S. Government Accountability Office, 2020). Even though rural areas account for 1/5 of the nation’s population, less than 10% of current practicing physicians are providing healthcare to these communities ( Jones *et al.*, 2009; McEllistrem-Evenson, 2011). Many of these rural areas are impacted by the lack of interest in primary care, aging practitioners, and various other factors ( Lakhan & Laird, 2009); leading to higher rates of death, disability, and chronic disease when compared to urban populations ( Doescher *et al.*, 2009; Jones *et al.*, 2009; McEllistrem-Evenson, 2011). There have been approaches to address the discrepancy in rural healthcare providers, especially with the current physician shortage and less than 3% of current medical students interested in serving rural areas ( McEllistrem-Evenson, 2011; Rabinowitz *et al.*, 2011). Tactics range from the national level with financial incentives ( Rosenblatt *et al.*, 1996) to the local level by implementing pipeline programs to spark interest and exposure to healthcare ( University of California Office of the President, 2018). Studies have shown the two main factors that influence a physician’s decision to practice in a rural location are programs that target students from rural areas and where a physician completes their training ( Brooks *et al.*, 2002; Lee & Nichols, 2014; Rosenblatt *et al.*, 1996; Wilson *et al.*, 2009). However, the literature also indicates that for students from primarily underserved areas, such as rural regions who may be the first in their families to pursue a career in medicine, an important aspect is utilizing mentorship. Specifically, an important component of mentorship includes communication with a mentor as well as role-modeling such that mentees and mentors are able to share similar experiences and mentors can advise mentees on how to overcome challenges of living in underserved areas or being the first in their families to pursue medicine.

Many studies show that mentorship results in benefits for both the mentee and the mentor ( Pololi *et al.*, 2002). For example, junior physicians who received mentorship increased skill development, job satisfaction, and career development ( Feldman *et al.*, 2010; Palepu *et al.*, 1998; Tom *et al.*, 2019) while mentors who partook in programs also reported higher job satisfaction and increase in retention at their current institutions ( Steven *et al.*, 2008). Regardless of whether the mentorship received was formal or informal, it still had a beneficial effect on the overall job preparation that the mentees felt and helped them navigate through job promotions and tenure ( McGuire *et al.*, 2004). Moreover, these benefits of mentorship in rural areas can help to foster a continuous supply of rural medical students and residents who decide to remain in rural areas after training ( Lee & Nichols, 2014; Wilson *et al*., 2009) thus increasing the number of potential mentors. Access to health professionals who could serve as potential mentors, was listed as the most common and difficult barrier to overcome ( DeCastro *et al.*, 2014; Nimmons *et al.*, 2019); with factors such as race, gender, and number of mentors not significantly affecting the level of satisfaction of those who received mentorship ( DeCastro *et al.*, 2014; Feldman *et al.*, 2010). Overall, increasing the number of physicians in rural communities can minimize the physician shortage and also help by increasing the level of interactions between premedical students, residents, and physicians on both a professional and personal level.

Although there are previous studies on the impact of mentorship including: 1) retention/supplementation of rural physicians and 2) the importance of mentorship for residents and practicing physicians, studies on mentorship for premedical students are lacking. The current literature highlights that challenges to sustaining mentoring relationships include gender and cultural differences ( Ramani *et al.*, 2006; Osman & Gottlieb, 2018), and competing obligations ( Manuel & Poorsattar, 2021). Previously, scholars in the field have described the skills and characteristics of effective mentors ( Kvernenes *et al.*, 2021; Stenfors-Hayes *et al.*, 2011). Nonetheless, the topic of mentorship in medicine remains a contested topic as to the critical time periods for mentorship. Countless studies have shown when mentees are paired with mentors that they can identify with, both parties benefit greater than in a mentor relationship where there is a lack of similarity ( Castellanos & Gloria, 2007; Hall & Burns, 2009; Ortiz-Walters *et al.*, 2010; Ross *et al.*, 2016). Our study builds on the previous literature and aims to describe best practices for developing and sustaining mentoring relationships. This study targeted medical students and aimed to examine how the mentorship they had experienced impacted their path to medicine. Specifically, this study looks at what aspects of previous mentorship participants considered valuable and successful within different time periods of their medical school trajectory with the goal being to inform best practices for the development of a high-value mentorship program to benefit the future of premedical students in underserved regions such as rural communities.

## Methods

### Ethics approval

All procedures performed were in accordance with the ethical standards of the institution and with the 1975 Helsinki Declaration and its later amendments or comparable ethical standards. Study procedures and materials were deemed exempt by the University of California, Merced’s Institutional Review Board (Protocol # UCM2019-56, decision 05/06/2019). Since the study was exempt, informed verbal consent was obtained individually prior to the interview. All participants agreed to participate and to the use of chosen quotations to be used in any reports and publications. No identifying information was collected. 

### Reflexivity

The authors of the manuscript included five current medical students, one trained educational researcher, and a project manager trained in research methods. The five medical students all identify as the first in their family to attend medical school, and four of them were the first in their family to attend college. The trained researcher and project manager are also first-generation college students. All authors had experience attending school in rural regions. All of the authors had previous experiences as mentees or mentors. The five medical students who served as authors were participating in a six-week summer program under the guidance of the educational researcher. The five medical students were the ones in charge of conducting the participant interviews. They were trained by the academic researcher in basic research methods and interviewing. The criteria for participating in the study was that participants were in medical school and either grew up or trained in a rural community; thus, the interviewers and participants had established prior relationships to the study based on this common characteristic. All interviewers had identified mentorship to be an important factor in their career trajectory; thus, their perception and experience with mentorship were critical to the data analysis as it helped identify barriers and facilitators for quality mentorship that targets prospective first-generation medical students.

### Design

This study consisted of structured interviews with 10 current medical students. Data collection occurred over a three-week period from May 14, 2019 to June 07, 2019. Questions were designed to elicit various experiences respondents had with mentoring including their opinions on mentorship program design. The study employed a grounded theory methodological approach. 10 interviews were conducted, but only seven were included in the analysis because three interviewees mentioned they had never had any type of mentorship and made no mention of any informal interactions that were identified as informal mentorship. Interviewees were recruited through word of mouth, among peers, and via networking at a community service event. The only criterion of inclusion was for participants to be a current medical student. No demographic data was collected, and interviews were de-identified. The study design was guided by a grounded theory approach. The positionality of the medical students was key in the interpretation of the results to ensure that the team was focused on elements important to the success of medical school training.

### Data analysis

Interviews ranged in duration from 20 to 40 minutes and were conducted by phone, by video call, or in person, with the audio recorded and transcribed verbatim. In-person interviews were conducted at a location of preference by the participant, and only the interviewer and participant were present. The research team coded the data using inductive methods to analyze the data for patterns in participants’ experiences ( Hanson *et al*., 2018). Each research member created a coding framework for the data, and the research team jointly determined the coded findings ( Gatell *et al*., 2017). The initial set of codes were derived from the study’s research questions and supplemented with inductively derived codes. Joint coding was used both initially while the research team established an initial coding scheme, and periodically thereafter to ensure continued intra- and inter-coder reliability. The joint coding consisted of all the authors reading through the data independently to develop the codes. Together the team then did a compare and contrast analysis to agree upon codes. Any discrepancies were discussed and reconciled during team meetings. The process for reconciling any discrepancies entailed reading through the data together and then discussing as a group of the coding. Parent codes were the broader categories or codes that emerged from the data while the child codes were identified as more specific examples of the parent codes. Microsoft Excel 2020 was used to store and organize the codes.

## Results

**Figure 1.**
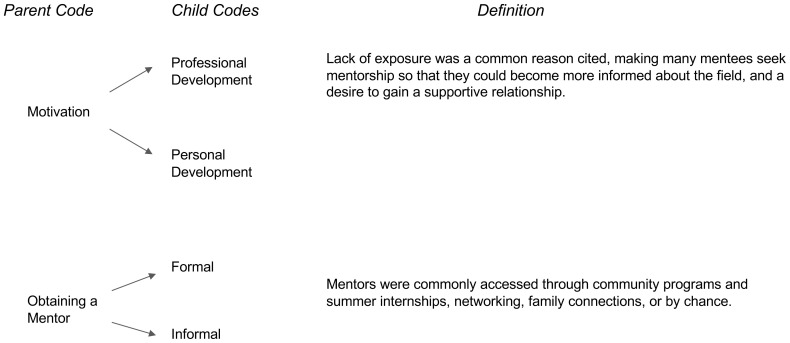
Before Obtaining a Mentor.

**Figure 2.**
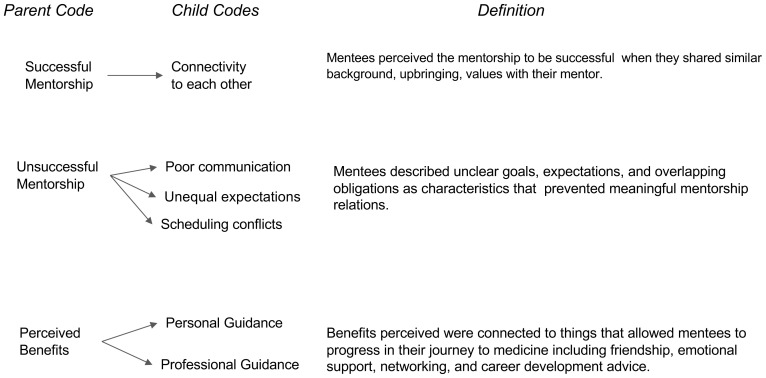
During the Mentorship Phase.

**Figure 3.**
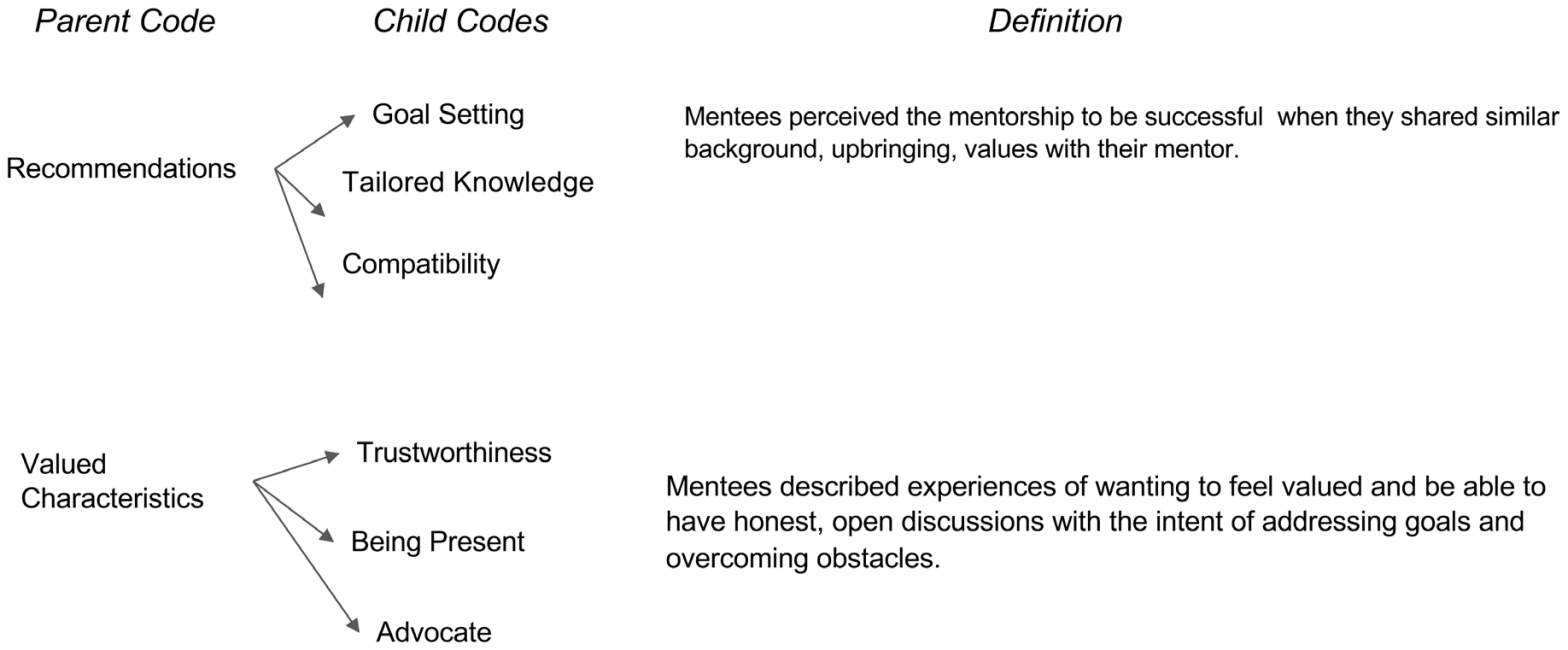
After the Mentorship.

Our study aims to describe best practices for developing and sustaining mentoring relationships. The analysis revealed three major time periods within medical students’ perception of the impact of mentorship on their personal and professional development to practice medicine, and specifically medicine within a rural area. The first major period was “Before Obtaining a Mentor” which entailed characteristics of the motivation behind obtaining a mentor and how the mentee went about obtaining their mentor ( Figure 1). The second major period of “During the Mentorship Relationship” entailed concepts of successful relationships, unsuccessful relationships/barriers, and benefits of the student’s relationship with their mentor ( Figure 2). The third major period was defined as “After the Mentorship” which focuses on the missed opportunities and recommendations ( Figure 3). These time periods and characteristics of each are presented below in sequential order.

### Before obtaining a mentor

***Motivation.*** Many participants reflected on similar prerequisites and lack of knowledge or experience along the pre-medical pathway as there did not seem to be “any *exposure to anything like medicine*” (Participant 1). Most students shared similar motivations as to why they sought mentorship, with many being driven by the desire for personal and professional growth. For example, one participant shared, “ *really just seeking out advice and what else can I do with the situation I kind of have*” (Participant 3). On a personal level, students reported desiring a mentor to be both “ *my motivation*” and role model who “ *can treat you like a whole person*” (Participant 1). More specifically, one student shared the need to “. *..being able to see students who were similar to me, or who struggled like me in the past*” Participant 6). Many students describe similar sentiments across the board regarding their motivation for personal development as future physicians. At the professional level, many students described the need to find inspiration and have a role model who would offer advice and help navigate the admissions requirements. For example, “ *[I was] motivated by the application itself. We do need letters of recommendation and for [those] to be strong letters, you do need to have some sort of relationship that goes beyond just the professor-student relationship*” (Participant 5). Our findings reveal that wanting exposure to people in medicine who reflected similar life experiences or values was common. Overall, students sought mentorship, both at personal and professional levels, in order to find and receive affirmative guidance in the field.

***Obtaining a mentor.*** The way students obtained mentorship varied from formal to informal settings. For example, one student shared how they obtained a mentor through a formal program, “ *[the program director] also brought in other people in the community who also served as mentors*” (Participant 5). Similarly, another student shared that they found a mentor through a “ *shadowing opportunity…[and] through alumni of my college*” (Participant 1). Informally, students were able to obtain mentors via networking, with one student saying, “ *sometimes it just happens by chance*” (Participant 2). More specifically, students provided examples of how they actively obtained mentorship through informal venues. A student shared, “ *[by]attending workshops I had a lot of direct exposure to the faculty, and because of that when they gave talks, I would just approach them after”* (Participant 4).

 Although the participants were able to obtain access to mentorship, there was a common theme in that students had limited opportunities to mentorship. There are many socioeconomic factors that may prevent students from accessing these programs, especially since these opportunities were often unpaid and had steep time requirements. For the participants in this study, they were oftentimes successful in obtaining mentorship through programs that made mentorship a part of their goal. They saw this as a formal manner to obtain mentorship. 

### During mentorship phase

***Successful mentorship characteristics.*** A prevalent theme was noted where the participants focused on discussing expectations on what qualities they valued in a successful mentor relationship. Student discussion focused on what aspects of their relationship with their mentor allowed them to classify the relationship as either successful or unsuccessful, as well as account what benefits they gained. Opinions on successful relationships often centered around shared qualities and life experiences between mentor and mentee, such as gender or ethnicity, which allow students to have deeper connections to their mentor and made them feel seen. As one student said, “ *They had a similar background as me and they faced some of the same struggles...similar mindsets...they really wanted to help other underrepresented students in pursuing a career in medicine*” (Participant 1).

***Unsuccessful mentorship characteristics.*** While unsuccessful relationships occurred for reasons opposite to successful relationships such as interpersonal and professional perspectives. For example, one student shared “ *different philosophies or ideas of medicine that kind of made [one] a little hesitant or not as comfortable...to communicate with them*” (Participant 7). This example seemed to be common across all participants as they felt that poor communication, unequal expectations, and difficulty scheduling were the main factors for an unsuccessful mentor relationship, raising the concern that poor mentorship experiences may not be due to individual mentors, but rather the lack of protected time set aside for individuals who wish to mentor. Another characteristic of unsuccessful relationships was the institutional barriers that may limit the interactions between mentors and mentees. For example, one student shared, *“[there are] barriers that come with being in a large institution where there are a lot of students who are in similar positions, kind of competing for the same mentors. There’s really no infrastructure to support a pre-med interaction with physicians*” (Participant 2).

***Perceived benefits.*** Benefits or gains from the mentorship relationship mirrored student motivation to obtaining a mentor in the *Before* phase. These fall into two categories: personal or professional guidance. On a personal level, many students discussed the difficulty of their journey to medicine, and the power their mentorship provided to fuel and encourage them to believe in themselves. For example, one student shared that having a mentor was like “ *a friendship and a connection that goes above and beyond medicine*” (Participant 1). Additionally, students discussed professional gains which reflected the arduous process of meeting the necessary requirements early on. As one student shared, *“[being] able to talk about and come up with strategies to do things differently, and [having someone who] advocated for you and helped you navigate the whole system*” (Participant 3) has proved to be a benefit of mentorship. Overall, the benefits perceived were based on the ability of a mentor to help the mentee progress towards their medical journey.

### After the mentorship

Lastly, discussion moved on to what characteristics they perceived to be of more value for future mentorship initiatives. Characteristics for future mentorships ranged from behaviors such as better communication to positivity and reliability. Specifically, students encourage mentors and mentees to set “ *more defining roles and communicating expectations*” (Participant 7) to achieve a successful and meaningful mentorship. Furthermore, students believe mentors ought to stay up to date on resources and requirements over time that are reflective of the mentee’s needs since the medical admissions process is constantly changing. For example, a student shared, *“[it is] useful to have some sort of training, especially for something [like] standardized as the medical school application. People may not be aware that things change over time*” (Participant 2). Formative pairing based on a compatibility survey early on to measure fit before beginning the relationship was also a theme seen that many believed would strengthen mentoring relationships. For example, a student shared, “ *do a survey and get an idea if it’s a good fit or not*” (Participant 5).

Overall, characteristics students list as valuable in a mentor include coming from diverse backgrounds and maintaining professional open communication. Some students valued openness as it “ *would make [them] feel more supported emotionally and professionally and personally*” (Participant 3). Students also valued support, positivity, and others described the importance of transparency and trustworthiness. Our findings revealed that in general mentees want to feel valued and be able to have honest, open discussions with the intent of addressing goals and overcoming obstacles.

## Discussion

This study primarily focused on identifying aspects of successful mentorship experiences in rural communities, where geographical isolation may limit exposure to medical professionals and/or established mentorship programs. Mentorship has been shown to play an important role in guiding people in the medical profession, starting from the undergraduate level and continuing into the postgraduate level. This study aimed to identify aspects of successful and satisfying versus unsuccessful aspects of mentorship relationships of medical students who grew up and/or trained in a rural setting. Specifically, the study findings include a deeper look at mentorship during different time periods of the students’ trajectory. Students found satisfaction and success in having close, open, and honest communication with their mentor, with reliable access, and a relationship of affirmation and role-modeling. That role-modeling stemmed from either shared community. Participants in this study either grew up or trained in rural communities and shared that at times their communities lacked extended resources and thus, having someone who had a similar background, or a shared community seemed to be an important aspect of a successful mentoring relationship. Concerning unsuccessful aspects, students found institutional barriers, lack of formal time commitment, and differences in personality, identity, and career to be particularly detrimental.

 Within the “ *After the Mentorship,”* students articulated the importance of opportunity, communication, and compatibility. Opportunities included those for success on the medical path while recommendations on communication were more nuanced. Students indicated that the lack of healthcare professionals not only impacted access to proper healthcare, but also impacted the opportunities presented to those interested in pursuing a healthcare career. In this case, students perceived the importance of opportunities that exposed students to the medical field. Furthermore, students felt communication between mentor and mentee ought to include clear expectations, as well as up-to-date knowledge, supportive honesty, and open approachability. Compatibility was emphasized, both personally and professionally, with one mentee even suggesting a measure of fit survey. Ultimately, these findings reveal the importance in being able to identify with the mentor and having a formal, structured program in place to help establish and strengthen the mentorship relationship. Based on these findings, we propose that for mentorship programs to create fulfilling, meaningful relationships between the mentor and mentee, there should be a focus on the following three components: 1) establishing expectations of both the mentee and mentor; 2) providing training on the current landscape for medical students; and 3) pairing of mentors and mentees based on similar backgrounds/life experiences.

### Establishing expectations for mentee and mentor

Programs, regardless of being formal and informal, should clearly establish the expectations of both mentors and mentees by ensuring that goals and timelines are set in place. Established expectations can aid in retaining mentors in rural who may oftentimes be overburdened by clinical duties. By ensuring basic guidelines such as educational, personal, and professional goals are created, allows both parties to build upon the base requirements comfortably without worrying about overstepping boundaries. Having these expectations reviewed and transparent would create an environment where mentees enter the program being able to navigate their roles as well as being aware of the expectations and will have a clear outline for receiving the most benefits from that relationship. Additionally, communication needs and expectations should also be addressed in program guidelines so that mentees can comfortably reach out to their mentors and understand how to properly address mentors and other professionals in a timely, professional manner, allowing a more robust and tailored mentorship experience. 

### Providing training on the current landscape for medical students

Another factor that we found in our research was that many felt that their mentors were not aware of the changes in medical school and residency program requirements. By having mentors learn about the changes in the medical school and/or residency application process and requirements, it would allow mentors to properly advise mentees and understand what experiences, advice, or resources they can provide to help ensure the mentee’s development.

### The pairing of mentors and mentees based on similar backgrounds/life experiences

It is not common practice to pair mentors with mentees that have similar backgrounds; with ethnic identity, socioeconomic status, gender identity, and cultural identity being a few principal examples. This pairing process is particularly important for students who oftentimes lack proper guidance or may not have access to mentors in rural communities. By incorporating a process that allows mentors and mentees to be paired together based on similarities, such as measuring the compatibility between the mentor and mentee through surveys or a matching process. Doing so increases the chance of retention between mentor and mentee within the program, but also beyond the program, allowing for a longitudinal mentorship experience. Studies have shown that providing input from both parties in the matching process, it allows for better mentorship outcomes and a willingness to understand the information being presented by the mentor.

Through the implementation of these components, we can increase the efficacy of mentorship and provide longer lasting relationships. Although there is an initial time intensive incorporation period that will have to occur to develop such a structure, the benefit to the mentee and mentor is exponentially increased. With access to mentorship programs already being a significant barrier to many mentees, we must ensure that once they are able to obtain formal mentorship, that the program is able to reward their efforts by providing a robust mentorship curriculum and structure. In addition, extended benefits of successful mentorship programs can help increase the pipeline of future healthcare professionals who will be more interested in mentoring others.

## Limitations of research

This study offers a look into the specific insights on mentorship in California’s San Joaquin Valley, although there were some limitations. The study includes a small sample size of medical students in a rural region. Additionally, we did not collect any identifying or demographic information from the participants to ensure confidentiality. Future research should include a larger sample size and should consider breaking down the findings by including demographic information (e.g. gender). Also, this study only explores the concept of mentorship from the mentees’ perception. Future research should also explore the mentors’ perceptions and experiences. Lastly, taking a quantitative approach to measuring satisfaction and outcomes of mentorship experiences should be considered.

## Data Availability

Due to privacy concerns, participants were informed that no identifiable data would be made to anyone outside of the research team who conducted the original study. Deidentified data will therefore only be provided upon request. Data requests can be made by contacting the corresponding author, Rosa D. Manzo, Ph.D. ( rmanzo3@ucmerced.edu). figshare: Interview Protocol_Mentorship.docx. https://doi.org/10.6084/m9.figshare.19103567.v1. Data are available under the terms of the Creative Commons Attribution 4.0 International license (CC-BY 4.0).
