# Supplementary material for: Medical mentorship deconstructed: an analysis and structural recommendation for high value mentorship
Source: MedEdPublish (2016). 2023 Aug 30;12:13. Originally published 2022 Mar 1. [Version 2] doi: 10.12688/mep.18944.2 (PMC10474344; doi:10.12688/mep.18944.2)
Supplement: Peer review history

# Peer review: Reviewer response for version 2

**DOI:** 10.21956/mep.21163.r34715

**Published:** 2023-09-05

**Authors:** Joanne Harris

**Affiliations:** 1 Medical School, The University of Buckingham, Buckingham, England, UK

Thank you for considering the comments made by myself and other reviewers and making changes to this article. This article now has an enhanced rationale and a much clearer flow. You have added additional literature in the introduction (although it would useful to refer back to some of this in the discussion). You have provided an ethics rationale that allows you to publish this data alongside unidentified quotes of your participants. I agree with a previous reviewer that if you are suggesting a change for future research in the limitation section, say why this would have been useful. What would collecting demographic data tell you. How would quantitative date help you to answer your research question?

 However this now reads as a good qualitative research project on a relevant topic.

Have any limitations of the research been acknowledged?

Partly

Is the work clearly and accurately presented and does it cite the current literature?

Partly

If applicable, is the statistical analysis and its interpretation appropriate?

Partly

Are all the source data underlying the results available to ensure full reproducibility?

No

Is the study design appropriate and is the work technically sound?

Partly

Are the conclusions drawn adequately supported by the results?

Partly

Are sufficient details of methods and analysis provided to allow replication by others?

No

Reviewer Expertise:

Professional identity formation of medical students and how this is affected by widening participation and cultural discrimination (particularly gender and race and ethnicity)

I confirm that I have read this submission and believe that I have an appropriate level of expertise to confirm that it is of an acceptable scientific standard.

# Peer review: Reviewer response for version 2

**DOI:** 10.21956/mep.21163.r34716

**Published:** 2023-09-01

**Authors:** Megan Grayce Anakin, Natalie Fleming

**Affiliations:** 1 Education Unit, Dunedin School of Medicine, University of Otago, Dunedin, New Zealand; 2 University of Otago, Dunedin, New Zealand

Thank you for taking the time to respond our review feedback and work to address the key suggestions we provided. The additional literature in the introduction shows how your study builds on existing literature. The reflexivity statement now provides more information about the authors that is relevant to reader for understanding how the data were analysed. The added details in the data analysis section describe and explain the joint coding process of the data analysis. Distinguishing quotations used from the various participants increases the trustworthiness and credibility of the findings. The revised Limitations section could offer an explanation about how an increased sample size in a future study might address a problem or concern with the sample analysed in this study. Likewise, the need for demographic information could also be explained in terms of how it might help the reader better understand the characteristics of the study participants and how their gender, ethnicity, age, and other relevant factors might impact their views and experiences of mentorship. The reader is also left wondering why the final sentence of the article suggests the use of a quantitative approach to measure satisfaction with mentorship experiences or other outcomes. An explanation of how this approach would build on the author’s findings would be helpful to understand the motivation for this future direction for their research.

Have any limitations of the research been acknowledged?

No

Is the work clearly and accurately presented and does it cite the current literature?

Partly

If applicable, is the statistical analysis and its interpretation appropriate?

Not applicable

Are all the source data underlying the results available to ensure full reproducibility?

Yes

Is the study design appropriate and is the work technically sound?

Partly

Are the conclusions drawn adequately supported by the results?

Partly

Are sufficient details of methods and analysis provided to allow replication by others?

No

Reviewer Expertise:

NA

We confirm that we have read this submission and believe that we have an appropriate level of expertise to confirm that it is of an acceptable scientific standard.

# Peer review: Reviewer response for version 1

**DOI:** 10.21956/mep.20301.r33019

**Published:** 2023-03-21

**Authors:** Joanne Harris

**Affiliations:** 1 Medical School, The University of Buckingham, Buckingham, England, UK

Thank you for asking me to review this article. This is an important topic about how mentorship is viewed by medical students and it is particularly good to see that this research has been carried out by medical students. However I have a few comments about how this research and the write up could be improved. There seems to be a confusion about whether the researchers are writing about how to encourage doctors to stay in rural areas, or the value of mentorship for 1st generation medical students (as the researchers identify themselves). I feel I am being made to assume that the San Joaquin valley is rural and deprived, but I have not been told this?

**Abstract**

 Largely well written, but more needs to be made of the fact that this is focussed on studying medical students in rural areas, if this was in fact the case. For example, this sentence 'The purpose of this project was to gain an understanding of the mentorship received by practicing medical students' does not fit with the introduction below which is focussing on rural areas, or the discussion 'This study primarily focused on identifying aspects of successful mentorship experiences in rural communities' as the selection criteria for students did not specify the students came from rural communities.

**Introduction**

 A good exploration of the literature, but could expand so not just related to USA? Australia and New Zealand would also have an interest in the rural aspects of this study? Again there seems to be some confusion as this section ends with the aim of this study being 'how the mentorship they had experienced impacted their path to medicine.'

**Methods**

 This area has some limitations as the reader is unsure of the selection criteria, the demographics of the participants and what was actually done. 

*Ethics.* I see that this study was deemed exempt from full ethics approval, but these type of questions asked of students for research and not for evaluation purposes normally would require an ethical oversight? For example did the participants give permission in their verbal consent for quotations to be made in a written study? Did you consider what you would do if the participants revealed an incidence of bad practice?

 I like the reflexivity section and it is good this is included, but for true reflexivity what is the link between first generation medical student researchers and sentence 'the only criterion of inclusion was for participants to be a current medical student' ? Interestingly this contradicts a sentence in reflexivity which says participants also could be graduates from medical school. Please check this.

**Design**

 Again there is confusion between rural, widening participation and all medical students. If this was selection by word of mouth were these your friends who you interviewed? In the discussion we learn 'Participants in this study either grew up or trained in rural communities ' but this is not part of the selection criteria where they only needed to be a current student. The lack of demographic data is a loss here. It is a shame you excluded three students who did not have a mentor as some of their views as to 'why not' may have also been useful to hear.

**Results**

 This is a strong section and I like the division into three time periods. You have selected relevant quotes from your students. However I realise you have not defined what a mentor is and how does this differ to a role model? Some of the expectations of the mentor e.g. to write references for them is taken up in some medical schools by the personal tutor system. Did these students have a personal tutor in addition to the mentor?

**Data analysis**

 It has become common place to say grounded theory was used to analyse the data. However if this is the case then this sentence cannot be true, 'the research team coded the data using a combination of deductive and inductive methods to analyze the data for patterns in participants’ experiences' as grounded theory only includes inductive data analysis.

**Discussion**

 This section returns again to a focus on rural communities where this is missing from the methods and data analysis. This is more of an additional data analysis section and would benefit from drawing on the literature again to back up your claims. Having said that I think you made some reasonable recommendations in this section. 

 Missing word in this sentence? 'Students indicated that many the lack of health care professionals'. 

 'oftentimes' is slang.

 I enjoyed reading this paper although it does read as a collection of individuals rather than a collaborative effort possibly with different people writing different sections? It is lacking an overarching message. Please have another go, trying to clearer about what you are aiming to achieve and how you have gone about this particularly with your study population. Thank you.

Have any limitations of the research been acknowledged?

Partly

Is the work clearly and accurately presented and does it cite the current literature?

Partly

If applicable, is the statistical analysis and its interpretation appropriate?

Partly

Are all the source data underlying the results available to ensure full reproducibility?

No

Is the study design appropriate and is the work technically sound?

Partly

Are the conclusions drawn adequately supported by the results?

Partly

Are sufficient details of methods and analysis provided to allow replication by others?

No

Reviewer Expertise:

Professional identity formation of medical students and how this is affected by widening participation and cultural discrimination (particularly gender and race and ethnicity)

I confirm that I have read this submission and believe that I have an appropriate level of expertise to confirm that it is of an acceptable scientific standard, however I have significant reservations, as outlined above.

# Peer review: Reviewer response for version 1

**DOI:** 10.21956/mep.20301.r33018

**Published:** 2023-03-17

**Authors:** Sireeshabala Arja

**Affiliations:** 1 Clinical Skills and Medical Education, Avalon University School of Medicine, Curacao, The Netherlands

Thank you for letting me to review this manuscript. 

**Introduction**

 The authors tried to let the readers know about the physician shortage, especially in rural areas, but it is difficult to understand the relationship between mentorship and the physician shortage in rural areas. I realized it when I read the discussion section, and the authors mentioned role modeling. The authors could modify the introduction following the problem-gap-hook model. 

**Methodology**

 The research methodology is appropriate. It would be interesting to know the demographics of the participants. I'd like to know the criteria or reason for recruiting ten participants. Did the authors reach saturation once they did seven interviews?

**Data analysis**

 As authors did Grounded theory analysis, did they employ compare and contrast analysis? It would be good to see more details regarding data analysis.

**Conclusions and discussion**

 The conclusions were drawn based on the results.

Have any limitations of the research been acknowledged?

Yes

Is the work clearly and accurately presented and does it cite the current literature?

Partly

If applicable, is the statistical analysis and its interpretation appropriate?

Partly

Are all the source data underlying the results available to ensure full reproducibility?

Yes

Is the study design appropriate and is the work technically sound?

Partly

Are the conclusions drawn adequately supported by the results?

Yes

Are sufficient details of methods and analysis provided to allow replication by others?

Partly

Reviewer Expertise:

Assessments, Accreditation/Regulation, Faculty Development, and Curriculum Development

I confirm that I have read this submission and believe that I have an appropriate level of expertise to confirm that it is of an acceptable scientific standard, however I have significant reservations, as outlined above.

# Peer review: Reviewer response for version 1

**DOI:** 10.21956/mep.20301.r32621

**Published:** 2022-10-18

**Authors:** Megan Grayce Anakin, Natalie Fleming

**Affiliations:** 1 Education Unit, Dunedin School of Medicine, University of Otago, Dunedin, New Zealand; 2 University of Otago, Dunedin, New Zealand

**Abstract:**

 Please consider revising the first sentence to that is does not read as an absolute statement. Consider signposting that the rural context is central to this study by adding the words ‘such as rural’ to the second sentence after the phrase: “In medically underserved areas,”. In the first sentence of the conclusion, please revise the final clause that states, “...that can be improved upon and universally changed” because the study was designed to “gain an understanding of mentorship” and not study improvement or universal change.

**Introduction:**

 The problem (I.e., doctor shortages in rural areas) introduced in the first paragraph is not sufficiently developed to provide the warrant for mentorship to be its solution. Please consider reducing the description of the problem and provide more information about why mentorship could address this problem. Likewise, please emphasise the connection between mentorship and rural practice in the second paragraph to strengthen the warrant for your study. Please define the term ‘high value mentorship’ for the reader. In the third paragraph, please revise the sentence beginning, “The literature fails...” by taking a critically appreciative approach by outlining that you are building upon the previous efforts of others. Please describe possible ‘critical time periods for mentorship’ and explain why they may be contested to support the warrant for the aim of your study. Please state the aim at the end of the introduction succinctly and align it with the aim statements made in the abstract and discussion. Please ensure that the rural context is included in the aim since it is featured in the introduction and discussion.

**Methods:**

 To strengthen the reflexivity statement, please add your team’s experience with education research, describe your conceptions of mentorship, and rural education background. The information about first-generation medical students needs further explanation, otherwise, the purpose is unclear as to how it relates to the rural education focus of this article. The reader needs to know this information in order to understand how the interview data were interpreted by the research team. An important limitation of this study was the exclusion of three interviews because “interviewees mentioned they had never had any type of mentorship” but, what if they described interactions that you might identify as informal mentorship? Another very important limitation is the lack of demographic information provided about the interviewees. This omission has an impact on the credibility of the results because the reader does not have any knowledge of the gender, age, ethnicity, year of programme, and experience in a rural education context of the study participants. Please remove repeated ideas in the design and data analysis paragraphs. Please describe who was involved in the joint coding, how coding discrepancies were reconciled, and how the parent and child codes, and themes were constructed.

**Results:**

 Please consider removing the first paragraph of the results because that information is repeated in the subsection and Figures that follow. Please consider revising the first sentence and state the overall results in relation to how they address the aim. Please consider omitting the Figures because they repeat information that is more clearly described in the subsections. If they are retained, please move them to appear below each group of results that they meant to illustrate. Please include a participant identification code with each quotation. This identification provides the reader with information to determine the trustworthiness of your results. It shows that all quotes do not come from one participant and that a range of response from participants are represented in your results. Another limitation of your study is in how the results were reported as predominantly unidirectional and mentor-dependent actions. As readers, we were left wondering about the role of the mentee in the mentorship relationship. Consider mentioning the mentee’s role in mentorship as an area for future study.

**Discussion:**

 Please move statements about mentorship and add references to support these claims to the introduction. Please move the information about the participant to the methods section. Consider rephrasing the aim statement to summarise your key findings as the introductory sentence of the first paragraph. Please revise discussion points that present your findings as absolute statements by softening your claims to show that they are your interpretation and are contestable. Discussion points should also be related to the literature so please add references to show the relevance and applicability of your findings to others. Please revise the limitations section to remove the comment about the small sample size because this is not the most important feature of your study design and other decisions you made had a greater impact on the trustworthiness and credibility of your findings.

 Please consider adding a conclusion containing 2-3 take home messages for the reader to contemplate.

Have any limitations of the research been acknowledged?

No

Is the work clearly and accurately presented and does it cite the current literature?

Partly

If applicable, is the statistical analysis and its interpretation appropriate?

Not applicable

Are all the source data underlying the results available to ensure full reproducibility?

Yes

Is the study design appropriate and is the work technically sound?

Partly

Are the conclusions drawn adequately supported by the results?

Partly

Are sufficient details of methods and analysis provided to allow replication by others?

No

Reviewer Expertise:

NA

We confirm that we have read this submission and believe that we have an appropriate level of expertise to confirm that it is of an acceptable scientific standard, however we have significant reservations, as outlined above.
